# Synchronous Luminal A Ductal Carcinoma In Situ in a Recurrent Benign Phyllodes Tumor Diagnosed Preoperatively: An Unusual Presentation of a Rare Neoplasm

**DOI:** 10.7759/cureus.23225

**Published:** 2022-03-16

**Authors:** Swagata Brahmachari, Meena Kumari, Tanya Sharma, Maheshkumar B Jagtap

**Affiliations:** 1 Surgery, All India Institute of Medical Sciences, Bhopal, Bhopal, IND; 2 General Surgery, All India Institute of Medical Sciences, Bhopal, Bhopal, IND; 3 Pathology and Laboratory Medicine, All India Institute of Medical Sciences, Bhopal, Bhopal, IND

**Keywords:** benign, emergency surgery, recurrent, breast carcinoma, phyllodes tumor, ductal carcinoma in situ

## Abstract

Phyllodes tumor (PT) is a rare benign tumor of the breast with a propensity to recur and metastasize. Ductal carcinoma in situ (DCIS) within PT is an extremely rare finding and is usually diagnosed postoperatively. We present a case of a 36-year-old female with a recurrent lump in her left breast diagnosed as a benign PT. Preoperatively, aspiration cytology revealed DCIS within the known case of recurrent PT. Emergency left modified radical mastectomy was performed due to an uncontrolled sudden hemorrhage from the tumor. Postoperatively, hormonal therapy was started based on immuno-histopathological findings.

DCIS in PT is not encountered frequently and hence no standard management protocol exists for such cases, but when detected by histopathology, the clinical management and prognosis have to undergo a complete change. Preoperative diagnosis and proper individualized management by a multidisciplinary team that ensures clear surgical margins and planned adjuvant therapy play a crucial role in preventing the recurrence of DCIS within PT.

## Introduction

Phyllodes tumor (PT) of the breast is an uncommon, fibroepithelial neoplasm, with an unpredictable behavior in the form of local recurrence and malignant transformation. Infrequent presentation poses diagnostic dilemmas and therapeutic challenges in this fast-growing neoplasm. Histologically, there is a biphasic proliferation of stromal and epithelial components, arranged in a leaf-like pattern, which gives it the name phyllodes [1]. PT accounts for only 0.3-0.9% of all breast tumors [1] and 2-3% of fibroepithelial breast tumors [2]. The World Health Organization (WHO) has classified PT based on histological characteristics of the stromal component into benign, borderline, and malignant subtypes, which helps in the prognostication of the clinical course and recurrence risk [3]. The stromal and ductal components of PT are usually benign but both can undergo malignant transformations, occurring mostly in the stromal component and very rarely in the epithelial component, in 1-2% of PT cases [4]. The coexistence of ductal carcinoma with PT is found in all subtypes of PT but is reportedly more common with malignant PT [5]. The presence of coexisting ductal carcinoma in situ (DCIS) within PT is clinically significant as it alters the prognosis and management. In this report, we describe a rare case of luminal A DCIS arising within a recurrent benign PT in a premenopausal patient, which posed a diagnostic challenge. The preoperative diagnosis of ductal carcinoma within PT helped to plan and surgically manage the breast and axilla in a single stage, and thus a second surgery was avoided. Our case report highlights the importance of histological assessment of PT with concurrent carcinomatous involvement preoperatively, for simultaneous planning of the management of both the pathologies as per standard guidelines. To the best of our knowledge, there is only one published case report in the literature that documents the subsequent development of luminal A DCIS following a previous resection of low-grade benign PT [6].

## Case presentation

**Figure 1:**
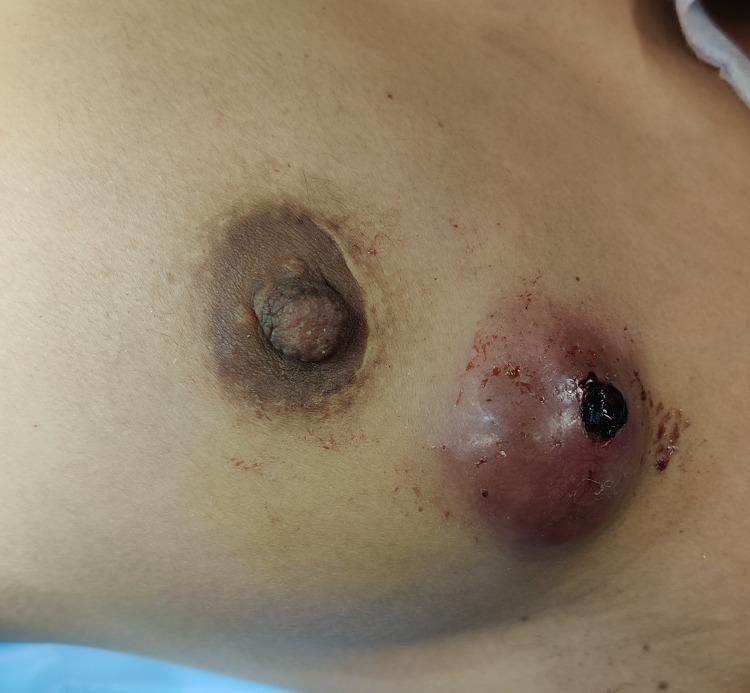
A breast lump of 5 x 5 cm in size in the upper outer quadrant of the left breast The lump was 1 cm away from the periareolar scar mark from previous surgery. The overlying skin was indurated and bleeding from the site of the core cut biopsy seen in the central portion

A multiparous, premenopausal woman aged 36 years presented with a gradually increasing left breast lump with pain for six months; there was no complaint of nipple discharge. The patient had a history of a similar lump in the same site two years back, which had been surgically excised and histologically diagnosed as benign PT. There was no family history of breast lump or carcinoma. On examination, a 5 x 5-cm, oval, firm, mildly tender breast lump with restricted mobility was observed at 1-2 o’clock, in the upper outer quadrant of the left breast, 1 cm away from the periareolar scar mark from the previous surgery. The overlying skin was adherent and indurated in the central portion. Discrete small axillary lymph nodes were palpable in the left axilla (Figure 1). The contralateral breast was normal.

**Figure 2:**
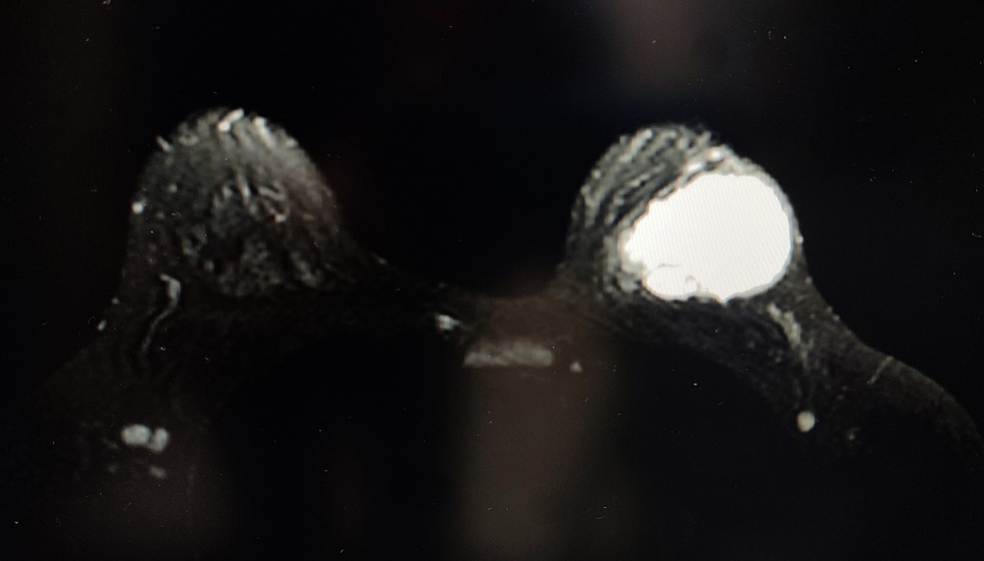
MRI breast with contrast The image reveals a large BI-RADS 4 lesion in the left breast, which is a heterogeneously enhancing mass with irregular shape and margins favoring phyllodes tumor with thinned out overlying skin and a scar over 2-3 o'clock and axillary lymph nodes MRI: magnetic resonance imaging; BI-RADS: Breast Imaging Reporting and Data System

Radio-imaging studies were done during the triple assessment. Sonomammography revealed a well-defined Breast Imaging Reporting and Data System (BI-RADS) 4 breast lump, which was 4 x 3 cm in size in the upper outer quadrant at the 12-1 o'clock position. MRI mammography showed a BI-RADS 4 lesion in the left breast with moderate internal vascularity but no calcifications, thinned-out skin over the lump, and preserved retro-mammary plane. The left axilla showed multiple small axillary lymph nodes (Figure 2).

**Figure 3:**
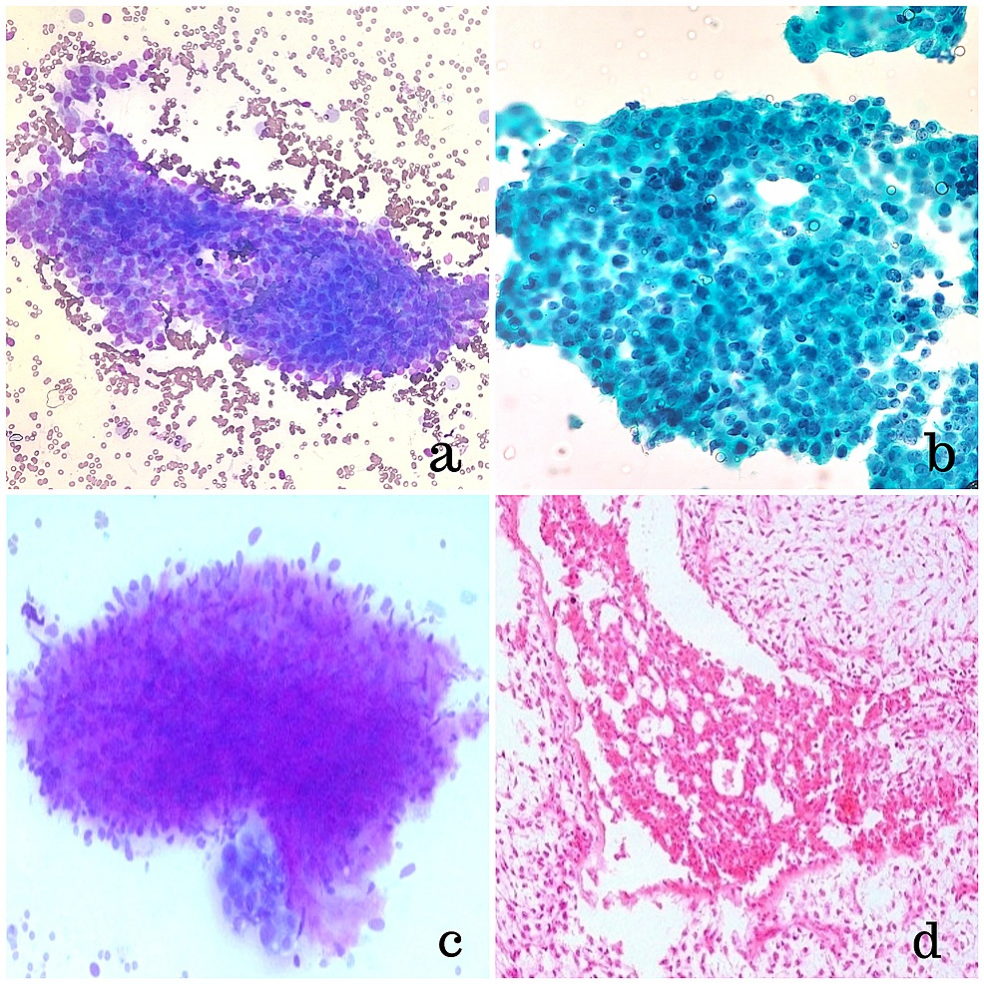
a, b: fine-needle aspiration cytology smear showing clusters of ductal epithelial cells displaying mild to moderate anisonucleosis, nuclear overlapping, and hyperchromasia with sparse myoepithelial cells (a: 20X MGG, b: 40X Papanicolaou); c: cellular stromal fragment (40X MGG); d: histopathology showing focus of low-grade ductal carcinoma in situ (20X H&E)

Image-guided fine-needle aspiration cytology (FNAC) of the left breast lump revealed a C4 lesion, raising suspicion for malignancy. A few clusters of ductal epithelial cells with a moderate anisonucleosis, nuclear overlapping, and hyperchromasia with a sparse number of myoepithelial cells were seen, suggestive of ductal carcinoma. Hence, a provisional diagnosis of ductal carcinoma within a known case of recurrent benign PT was made (Figure 3).

A core cut biopsy was performed for confirmation but the procedure was complicated by profuse tumoral bleed. The decision to perform an emergency modified radical mastectomy was taken as the provisional diagnosis was a recurrent benign PT with ductal carcinoma with axillary lymphadenopathy.

**Figure 4:**
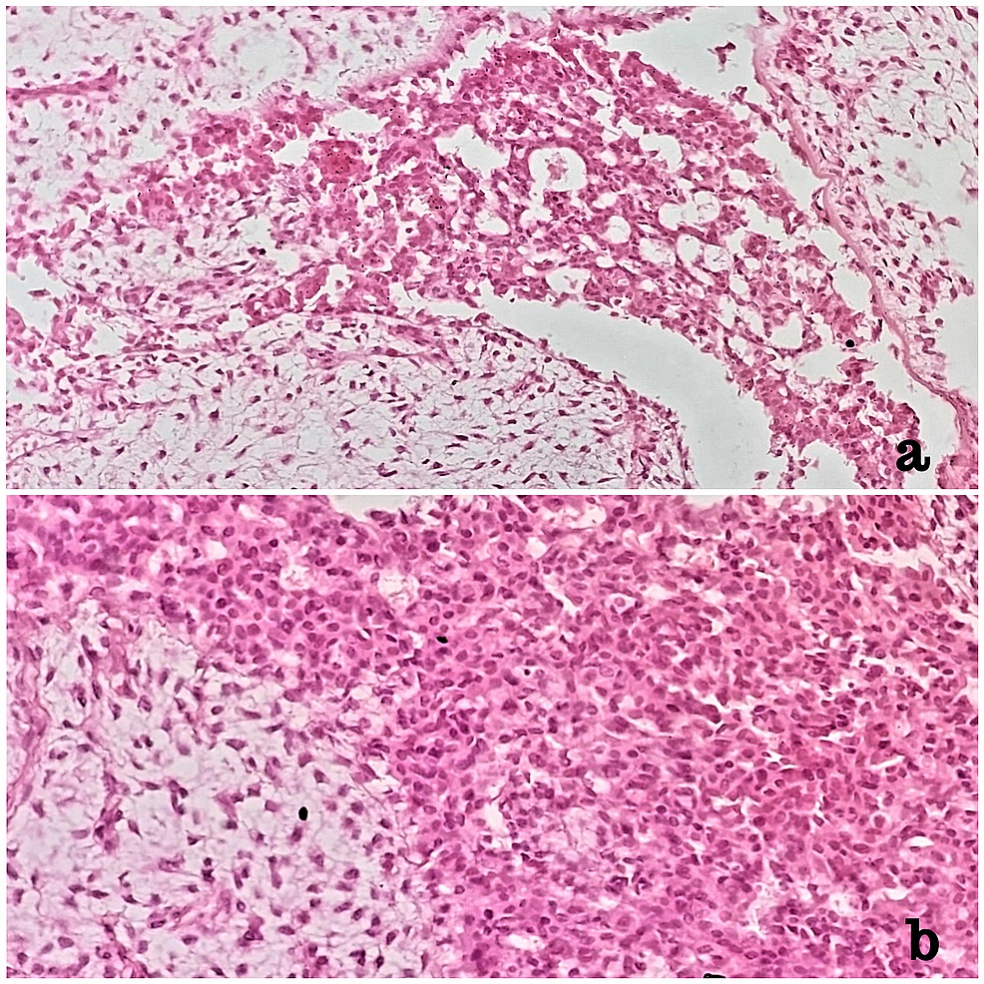
Histopathology showing a focus of low-grade ductal carcinoma in situ arising within the epithelial component of phyllodes tumor (a: 20X, b: 40X, H&E)

During the histopathological examination, the cut section showed a circumscribed encapsulated tumor measuring 4.5 x 4.0 x 4.0 cm in the upper outer quadrant of the left breast with slits and clefts typical of phyllodes. Microscopic examination revealed a benign fibroepithelial tumor with the proliferation of stromal as well as glandular components. The stromal component exhibited foci of stromal overgrowth with moderate atypia, variable cellularity with areas of myxoid change, and mitotic activity of less than 5 per 10 high-power fields, suggestive of benign PT. The glandular component showed small areas of ductal epithelial hyperplasia with few foci of low to intermediate-grade DCIS arranged in cribriform and solid pattern (Figure 4).

Tumor cells were seen protruding through the ulcerated skin, but margins were found to be tumor-free, with no evidence of invasive carcinoma. Thus, the breast lump was diagnosed as luminal type A DCIS (ER/PR+/Her2 neu -ve, Ki67 5-10%) within recurrent PT (TisN0M0). Based on the University of Southern California/Van Nuys Prognostic Index, our patient had low to intermediate-risk DCIS. As per the tumor board’s decision, the patient was given adjuvant hormonal therapy (tamoxifen 10 mg); she is currently under regular follow-up and has shown no clinical or radiographic evidence of disease progression.

## Discussion

PT is diagnosed and graded as per the WHO classification of tumors of the breast based on histological characteristics of a stromal component of PT, such as overgrowth, hypercellularity, atypia, high mitosis rate, infiltrative borders, and the presence of necrosis, into benign, borderline, and malignant subtypes [3]. The diagnosis becomes challenging when there is a coexisting carcinoma with PT. The carcinomatous transformation of the epithelial component of PT may present as in situ or invasive, ductal, or lobular breast carcinoma, in a background of stromal neoplasms in all subtypes of PT. It may present within the PT or as a separate lesion near the PT, in the ipsilateral or contralateral breast [5,7]. Moreover, a coexisting DCIS within PT has to be differentiated from carcinosarcoma (a subtype of metaplastic carcinoma) where epithelial markers are absent [2]. Hence, the diagnosis of coexisting DCIS in PT is made after histological subtyping of PT and assessment of coexisting DCIS by immunohistochemistry.

The pathogenesis of synchronous carcinoma within PT is still unknown. Histologically, PT arises from the periductal stromal cell whereas carcinomas originate from ductal or lobular breast tissue [8]. Molecular, cytogenetic, and immunohistochemical examination of PT tissues has shown an epithelial-stromal interdependence with genetic and oncogenic mutations in both epithelium and stroma, suggesting that both epithelium and stroma can undergo neoplastic changes [5,8,9]. Stimulation of the epithelial component of the PT locally by hormones and systemic growth factors or by the stromal component of the PT can explain the increased coexistence of higher-grade DCIS within malignant PT compared to benign PT [5,9]. The reported case involved DCIS in recurrent benign PT.

Co et al. have suggested that synchronous breast carcinoma with PT is under-detected and underreported [5]. A review of the literature by the authors on published cases of coexisting breast carcinoma within PT in the English language in indexed journals till 2021 revealed 25 such cases, of which only 18 cases were of pure DCIS within benign PT. Only one case of DCIS in recurrent benign PT has been reported, by Nio et al., which is similar to this case [6]. To the best of our knowledge, Leong and Meredith first reported a case of invasive ductal carcinoma arising within a recurrent benign PT in 1980 [10]. The details of the published cases of coexisting DCIS within recurrent PT are summarized in Table 1. In the reported case, DCIS was found within a recurrent benign PT but diagnosed preoperatively, and hence it is probably the first such case to be reported.

**Table 1:** Published cases of breast carcinoma within recurrent benign PT PT: phyllodes tumor; BPT: benign phyllodes tumor; R: recurrent; IDC: invasive ductal carcinoma; DCIS: ductal carcinoma in situ; ER: estrogen receptor; PR: progesterone receptor; W: within; MX: mastectomy; WLE: wide local excision; SLND: sentinel lymph node dissection; ALND: axillary lymph node dissection

Serial number	Authors	Year	Patient age (years)	PT-benign PT		Carcinoma component (epithelial type)-DCIS			Location of Ca and PT	Management		
				Type	Size (mm)	Type	Size (mm)	IHC(+ve)	Within	Surgery	ALND/SLND	LN status
1	Leong and Meredith [10]	1980	51	BPT (R)	4	IDC			W	MX	SLND	Negative
2	Nio et al. [6]	2011	53	BPT (R)	3.5	DCIS	0.5	ER/PR	W	WLE	Not done	Not known
3	Present case	2022	36	BPT (R)	4.5	DCIS	2	ER/PR	W	MX	ALND	Negative

Demographic studies have found the median age of presentation of PT in western countries falls in the 40-50-year age group and DCIS with PT is found mostly in individuals above 50 years. In the Asian population, the primary PT is found in a much younger age group of 25-30 years, and PT with coexisting DCIS is found more commonly in patients below 40 years in age, as illustrated in our case [2,11]. Clinically, PT presents as a rapidly growing lobulated, painless breast mass with an average size of 4-5 cm, as seen in our case [11].

During the triple assessment, a preoperative diagnosis of coexisting DCIS within a PT is difficult as dominant PT occupies a larger area than DCIS, which does not usually form a mass, and there are no specific preoperative clinical or radiological indicators for this dual pathology [5]. Usually, this coexistence is discovered incidentally during the histopathological examination of the excised specimen after definitive surgery for PT [7]. Lui et al. in 2018 found a preoperative histological diagnosis of DCIS in one patient [12]. Saimura et al. (2018) performed a preoperative needle biopsy in all seven patients of carcinoma with a fibroepithelial tumor but could diagnose DCIS in PT in only one patient [13]. In this reported case of recurrent PT, carcinoma was detected preoperatively by image-guided FNAC. For evaluation and subtyping of the stromal component, a meticulous sampling is indispensable along with a search for any malignant epithelial transformation in the same lesion elsewhere. Also, radiologists and pathologists must be aware of the possibility of concurrent carcinoma with PT as it alters the prognosis and management from wide local excision to axillary management and adjuvant treatment.

The rarity and varied presentations of synchronous DCIS in PT limit the standardization of protocol for management and follow-up. The management of benign PT as per the National Comprehensive Cancer Network (NCCN) guidelines is wide local excision with ≥1-cm negative margin or simple mastectomy without axillary lymph node dissection [13]. Adjuvant radiotherapy is also not necessary in cases of benign PT with clear resection margins [14].

Preoperative diagnosis and pathologically clear surgical margins with adjuvant treatment play a crucial role in preventing recurrence of ductal carcinoma within PT. Synchronous breast carcinoma in PT is usually not diagnosed preoperatively, and hence routine axillary surgery is not done along with definitive surgical excision of PT. After the histopathological diagnosis of dual pathology of DCIS with PT, the treatment plan has to be revised and axillary lymph node evaluation and receptor studies have to be done as a second surgery for staging and planning for adjuvant treatment such as chemotherapy and hormonal and radiotherapy [5,15]. As carcinoma often presents in the early stage, surgery and adjuvant treatments provide a good prognosis. Based on the preoperative diagnosis of DCIS within PT with axillary lymphadenopathy, a modified radical mastectomy was done in the reported case. Adjuvant hormonal therapy (tamoxifen) was given after a definitive diagnosis of recurrent benign PT with luminal A DCIS. Luminal A type of breast carcinoma benefits from hormonal therapy. Receptor studies have shown ER/PR positivity in the majority of cases as in our case [5].

Metastasis is associated mostly with the hematogenous route in 15% of malignant PT and with the lymphatic route in only 1.1-3.8% of cases, and hence axillary lymph node dissection is not done, without axillary lymphadenopathy [15]. While formulating a management plan in cases of synchronous DCIS with PT, both the stromal and epithelial components should be evaluated and managed independently by surgical excision, axillary staging, and adjuvant therapy as per the immunohistochemical status [15]. Hence, a multidisciplinary approach is needed to individualize the treatment plan as per the pathological stage of both stromal and epithelial components, axillary nodal staging, margin status, and receptor status for surgical excision, axillary management, and adjuvant treatment.

One more peculiarity of our case is that it was operated on as an emergency, although breast surgery is rarely considered an emergency procedure. Only 10 cases of emergency breast surgery have been reported to date, and those were mostly done to control bleeding from PT or rarely breast carcinoma not responding to compression as in our case [16]. Local recurrence is characteristic of PT and can occur with grade progression even in benign PT. The incidence of local recurrence in benign PT was found to be 8% in 10 years without metastasis in the western population, but in the Asian population, Tan et al. found it to be 10.9%, emphasizing the need for regular yearly clinical and imaging follow-up [2,12].

The prognosis depends on the nature of carcinoma and PT, with DCIS having a more favorable prognosis than the invasive variants; however, it carries a risk of progression to invasive disease [11]. Benign PT and DCIS are generally associated with a more favorable prognosis than malignant PT and invasive carcinoma due to increased distant metastasis and local recurrence in the latter and no deaths have yet been reported [5,11]. The survival rate of PT patients even with the presence of coexisting carcinoma is excellent in DCIS with clear surgical margins.

## Conclusions

Clinicians, pathologists, and radiologists should be aware of the possibility of encountering a coexisting carcinoma component with PT, which is rare and under-diagnosed, but when detected by immunohistopathology, alters the clinical management and prognosis as per the type and stage of ductal carcinoma and PT. There is no standard management protocol for such cases; so, while formulating a management plan, both the stromal and epithelial components should be taken into account. Preoperative diagnosis and proper management by ensuring clear surgical margins and planned adjuvant therapy play a crucial role in preventing the recurrence of DCIS within PT. A multidisciplinary approach is needed to individualize the treatment plan as per the pathological stage of both stromal and epithelial components, axillary nodal staging, margin status, and receptor status for surgical excision, axillary management, and adjuvant treatment. Regular follow-up is also important as prognostic data for this rare lesion is limited.
